# Value of imaging examinations in diagnosing lumbar disc herniation: A systematic review and meta-analysis

**DOI:** 10.3389/fsurg.2022.1020766

**Published:** 2023-01-06

**Authors:** Zhihao Huang, Pengfei Zhao, Chengming Zhang, Jingtao Wu, Ruidong Liu

**Affiliations:** ^1^ School of Big Data and Fundamental Sciences, Shandong Institute of Petroleum and Chemical Technology, Dongying, China; ^2^ Department of Clinical Pharmacy, Weifang People's Hospital, Weifang, China; ^3^ School of Intelligent Manufacturing and Control Engineering, Shandong Institute of Petroleum and Chemical Technology, Dongying, China; ^4^ School of Physical Education, Leshan Normal University, Leshan, China; ^5^ Sports Coaching College, Beijing Sport University, Beijing, China

**Keywords:** Lumbar Disc Herniation, Magnetic Resonance Imaging, Computed Tomography, myelography, meta-analysis

## Abstract

**Purpose:**

To systematically review the clinical value of three imaging examinations (Magnetic Resonance Imaging, Computed Tomography, and myelography) in the diagnosis of Lumbar Disc Herniation.

**Methods:**

Databases including PubMed, Embase, The Cochrane Library, Web of Science, CBM, CNKI, WanFang Data, and VIP were electronically searched to collect relevant studies on three imaging examinations in the diagnosis of Lumbar Disc Herniation from inception to July 1, 2021. Two reviewers using the Quality Assessment of Diagnostic Accuracy Studies-2 tool independently screened the literature, extracted the data, and assessed the risk of bias of included studies. Then, meta-analysis was performed by using Meta-DiSc 1.4 software and Stata 15.0 software.

**Results:**

A total of 38 studies from 19 articles were included, involving 1,875 patients. The results showed that the pooled Sensitivity, pooled Specificity, pooled Positive Likelihood Ratio, pooled Negative Likelihood Ratio, pooled Diagnostic Odds Ratio, Area Under the Curve of Summary Receiver Operating Characteristic, and Q* were 0.89 (95%CI: 0.87–0.91), 0.83 (95%CI: 0.78–0.87), 4.57 (95%CI: 2.95–7.08), 0.14 (95%CI: 0.09–0.22), 39.80 (95%CI: 18.35–86.32), 0.934, and 0.870, respectively, for Magnetic Resonance Imaging. The pooled Sensitivity, pooled Specificity, pooled Positive Likelihood Ratio, pooled Negative Likelihood Ratio, pooled Diagnostic Odds Ratio, Area Under the Curve of Summary Receiver Operating Characteristic, and Q* were 0.82 (95%CI: 0.79–0.85), 0.78 (95%CI: 0.73–0.82), 3.54 (95%CI: 2.86–4.39), 0.19 (95%CI: 0.12–0.30), 20.47 (95%CI: 10.31–40.65), 0.835, and 0.792, respectively, for Computed Tomography. The pooled Sensitivity, pooled Specificity, pooled Positive Likelihood Ratio, pooled Negative Likelihood Ratio, pooled Diagnostic Odds Ratio, Area Under the Curve of Summary Receiver Operating Characteristic, and Q* were 0.79 (95%CI: 0.75–0.82), 0.75 (95%CI: 0.70–0.80), 2.94 (95%CI: 2.43–3.56), 0.29 (95%CI: 0.21–0.42), 9.59 (95%CI: 7.05–13.04), 0.834, and 0.767 respectively, for myelography.

**Conclusion:**

Three imaging examinations had high diagnostic value. In addition, compared with myelography, Magnetic Resonance Imaging had a higher diagnostic value.

## Introduction

Lumbar Disc Herniation (LDH) is defined as a localized displacement of disc material (nucleus, cartilage, fragmented apophyseal bone, annular tissue, or any combination thereof) (1). When the displayed disc material compresses the local nerve, there will be a series of symptoms such as low back pain, radiating pain on one or both sides of the lower limb, numbness, intermittent claudication, difficulty walking, and even muscle atrophy, which will seriously affect the daily life of patients. It is estimated that approximately 2%–3% of the population may be affected, with a prevalence of 4.8% among men and 2.5% among women older than 35 (2). Approximately 95% of herniated discs occur at the low lumbar spine (L4/5 and L5/S1 level) in people aged 25 to 55 (3). With the rapid development of the economy and society, people's way of life and work has changed. Patients with LDH show an increasing trend and tend to be younger. The treatment cycle of the disease is long, and the cost is high, resulting in a heavy burden on the family and society. Timely and accurate diagnosis plays an important role in the later treatment and rehabilitation of LDH.

Imaging examinations are often used in patients with low back pain and/or leg pain to assess the compression of a nerve root caused by disc herniation or spinal and cauda equina syndrome (4–7). Furthermore, imaging examinations can also be used to identify the clinical symptoms of affected disc levels before surgery (8). However, there are different reports on the accuracy of imaging examinations in the diagnosis of LDH, and there is a lack of multicenter and large-scale research. The purpose of this meta-analysis is to systematically review the published literature on the diagnosis of LDH by Magnetic Resonance Imaging (MRI), Computed Tomography (CT), and myelography through meta-analysis so as to provide a basis for clarifying the accuracy of imaging examination in the diagnosis of LDH.

## Materials and methods

This review followed the meta-analysis of Standards for Reporting of Diagnostic Accuracy Studies (STARD) 2015 guidelines (9) and was reported in accordance with the Preferred Reporting Items for Systematic Reviews and Meta-Analyses (PRISMA) 2020 (10). The review was registered in International Prospective Register of Systematic Reviews (PROSPERO) (registration number CRD42021269796).

### Literature search strategy

All relevant literature from eight databases, namely, PubMed, Embase, The Cochrane Library, Web of Science, CBM, CNKI, WanFang Data, and VIP, were explored from inception to July 1, 2021. To minimize the missing literature, the references in the included studies were traced to **supplement data**.

### Eligibility criteria

The inclusion criteria were as follows: participants with suspected LDH who underwent MRI, CT, or myelography before reference standard examinations (not limited by age, race, and nationality); prospective or retrospective study design; direct or indirect availability of the results—True Positive (TP), False Positive (FP), False Negative (FN), and True Negative (TN). The exclusion criteria were as follows: duplicate articles; reviews, conference abstracts, animal studies, and case reports; studies that did not describe specific diagnostic reference standards of LDH; studies with unclear measurement indicators, inappropriate statistical methods adopted, or important outcome indicators not fully explained; studies that were unable to obtain full text directly or indirectly; and studies not in English or Chinese.

### Literature screening, data extraction

Two reviewers independently screened the literature and extracted and cross-checked the data. In case of disagreements, a third party was consulted to assist in the judgment. During literature screening, first, the title and abstract were read. Then, after the exclusion of irrelevant literature, the full text was read to determine whether it was finally included. Data extraction mainly included the basic characteristics of the included studies, such as author, publication year, country, design type, sample size, diagnostic method, and reference standard. Results considered, such as TP, FP, FN, and TN.

### Risk of bias assessment of included studies

Two reviewers independently used the Quality Assessment of Diagnostic Accuracy Studies-2 (QUADAS-2) tool to evaluate the risk of bias of included studies (11). In case of disagreements, a third party was consulted to assist in the judgment. Each item was assessed as “yes” (low bias or good suitability), “no” (high bias or poor suitability), or “unclear” (lack of relevant information or uncertainty for the bias).

### Outcome indicators

These include pooled Sensitivity (Sen), pooled Specificity (Spe), pooled Positive Likelihood Ratio (+LR), pooled Negative Likelihood Ratio (−LR), pooled Diagnostic Odds Ratio (DOR), Summary Receiver Operating Characteristic (SROC), Area Under the Curve (AUC) of SROC, and Q*.

### Statistical analysis

Review Manager 5.3 software was used to evaluate the risk of bias of the included studies, Meta-DiSc 1.4 was used for meta-analysis, Stata 15.0 was used for sensitivity analysis, and publication bias test. First, Spearman's correlation coefficient between the logarithm of Sen and the logarithm of (1−Spe) was calculated to analyze the heterogeneity caused by the threshold effect: if the *P*-value of Spearman's correlation coefficient was less than 0.05, it indicated that there was heterogeneity caused by threshold effect. It was necessary to conduct a meta-analysis after adjusting and combining the confounding factors between studies and considering the interaction between Sen and Spe. If the *P*-value of Spearman's correlation coefficient was more than 0.05, it indicated that there was no heterogeneity caused by the threshold effect. The next step was to test the heterogeneity of the no-threshold effect (12, 13). *I*^2^ was used to analyze the no-threshold heterogeneity (14): if *I*^2^ < 50%, it indicated that there was little heterogeneity between studies, and the fixed-effects model was used for pooling. If *I*^2^ ≥ 50%, it indicated that there was great heterogeneity between studies. Meta-regression was used to find the potential factors causing heterogeneity (15), and then subgroup analysis was performed (16). If the source of heterogeneity could not be found, the random effects model was used for pooling. According to the corresponding model, calculated Sen_(pooled)_, Spe_(pooled)_, +LR_(pooled)_, −LR_(pooled)_, and DOR_(pooled)_; draw SROC; and calculated AUC and Q* of the included studies. Among them, the higher the Sen, Spe, DOR, Q*, and +LR, the lower the −LR, and the closer was AUC to 1, indicating the higher value of imaging examinations in diagnosing LDH; otherwise, the value was lower (13, 17–19). The stability of the research results was analyzed by sensitivity analysis. The included literature was excluded one by one, and then meta-analysis was performed again. The results were compared with those before exclusion. If the change was small, it indicated that the stability of the included literature was good and the results were credible. If there were significant changes, it indicated that the results were not credible (20). Finally, the publication bias was tested by Deek's funnel plot (21). If the *P*-value of the slope coefficient was more than 0.05, it indicated that there was no publication bias. On the contrary, it indicated that there was publication bias.

## Results

### The result of the literature search

**Figure 1:**
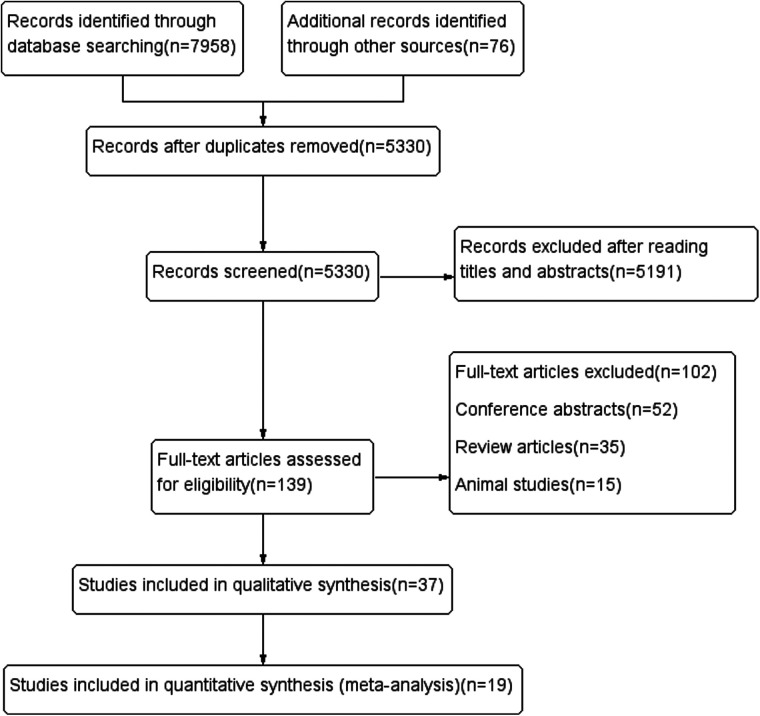
Flow diagram of literature search and selction process.

A total of 8,034 relevant articles were obtained. After the layer-by-layer screening, 19 articles (22–40) were finally included. The process of the literature search is shown in Figure 1 and Supplementary Method S1. Detailed information on the included literature is shown in Table 1.

**Table 1:** Characteristics of included studies.

Study	Country	Type of study	Reported outcome	Reference standard	Age (range)	Sample size (male/female)
Aejmelaeus 1984 (22)	Finland	Prospective	Myelography	Surgical findings	43.8 (14–82)	200 (109/91)
Bernard 1994 (23)	America	Prospective	MRI	Surgical findings	50 (23–74)	33 (20/13)
Birney 1992 (24)	America	Retrospective	MRI	Surgical findings	39 (20–71)	90 (48/49)
Bischoff 1993 (25)	America	Retrospective	Myelography;MRI	Surgical findings	20–79	57 (29/28)
Chawalparit 2006 (26)	Thailand	Prospective	MRI	Surgical findings	42.9 (21–60)	123 (61/62)
Firooznia 1984 (27)	America	Retrospective	CT	Surgical findings	49 (19–76)	100 (61/39)
Forristall 1988 (28)	America	Prospective	CT;MRI	Surgical findings	45 (22–74)	32 (25/7)
Gillström 1986 (29)	Sweden	Prospective	Myelography;CT	Surgical findings	23–74	37 (22/15)
Haughton 1982 (30)	America	Prospective	Myelography;CT	Surgical findings	13–72	107 (58/49)
Huang 2020 (31)	China	Prospective	MRI	Clinical data	51.9 (37–65)	161 (93/68)
Jackson 1989 (32)	America	Prospective	Myelography;CT	Surgical findings	42.7 (21–76)	124 (87/37)
Jackson 1989 (33)	America	Prospective	Myelography;CT;MRI	Surgical findings	39.6 (18–70)	59 (33/26)
Janssen 1994 (34)	America	Retrospective	Myelography;MRI	Surgical findings	46 (27–73)	60 (23/37)
Kamal 2009 (35)	Bangladesh	Prospective	MRI	Surgical findings	NA	40 (28/12)
Masaryk 1987 (36)	America	Prospective	MRI	Surgical findings	26–66	20 (13/7)
Modic 1986 (37)	America	Prospective	Myelography;CT;MRI	Surgical findings	46 (19–73)	48 (NA)
Mullin 2000 (38)	America	Prospective	MRI	Surgical findings	NA	28 (NA)
Schipper 1987 (39)	The Netherlands	Prospective	Myelography; CT	Surgical findings	43 (NA)	461 (NA)
Thornbury 1993 (40)	America	Retrospective	CT;MRI	Surgical findings	39.6 (21–72)	95 (61/34)

### Risk of bias assessment of the included studies

**Figure 2:**
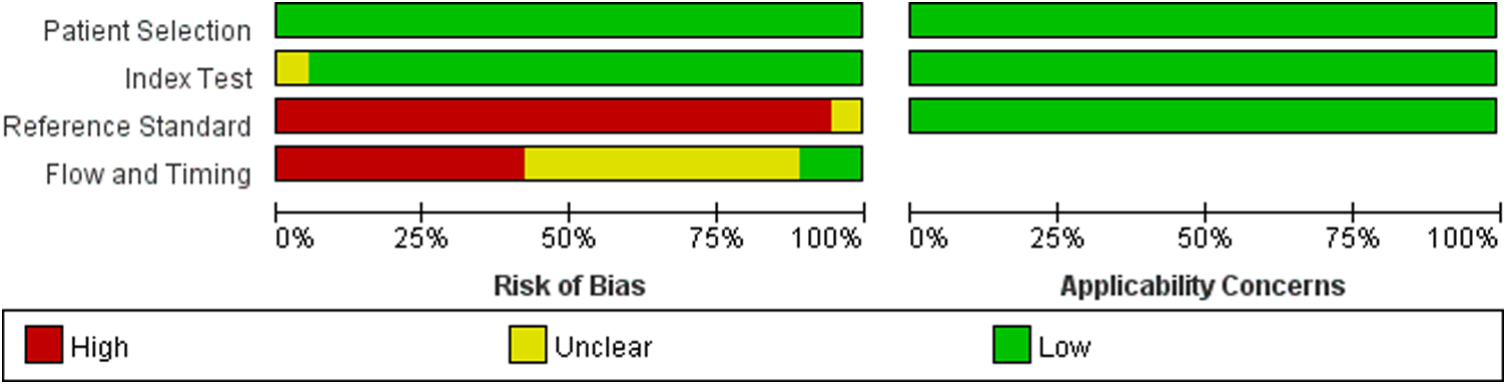
Risk of bias and applicability concerns graph.

**Figure 3:**
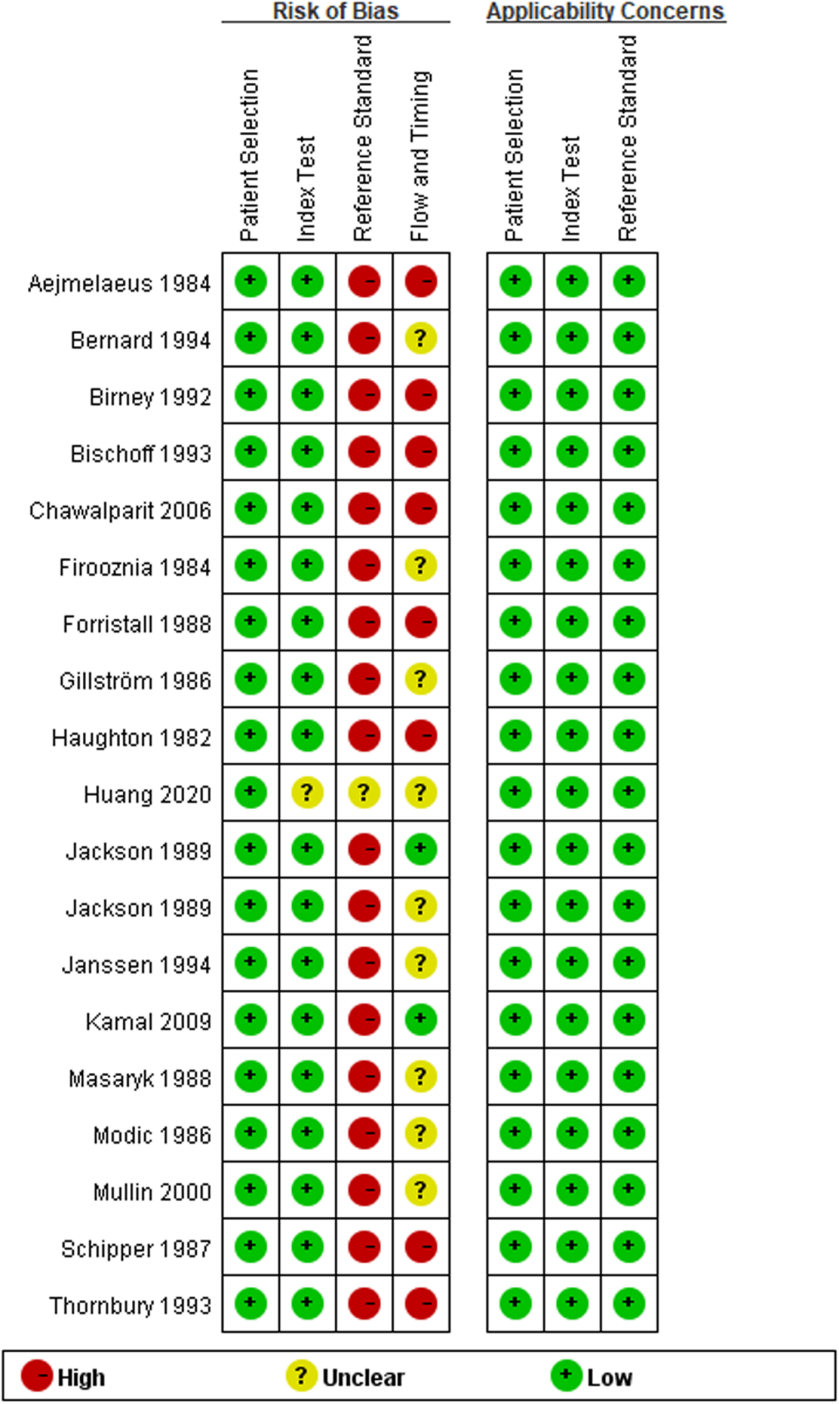
Risk of bias and applicability concerns summary.

The results of the QUADAS-2 tool showed that the implementation of diagnostic tests and the rationality of the reference standard included in this meta-analysis were of good quality, suggesting that the included studies had high quality and were less likely to cause selection bias (41). However, we were not satisfied with the reference standard. The main reason is that most studies regarded surgical findings as the reference standard, and all patients need imaging examinations before surgery. The detailed information is shown in Figures 2, 3 and Supplementary Table S1.

### Meta-analysis of MRI

Thirteen articles with 19 studies were included (Table 2).

**Table 2:** Characteristics of MRI diagnostic tests.

Study name	TP	FP	FN	TN
Bernard 1994 (23)	33	9	13	11
Birney 1992 (24)	70	0	5	1
Bischoff 1993 (25)	25	10	10	27
Chawalparit 2006 (26)	19	2	4	8
Chawalparit 2006 (26)	19	3	4	7
Forristall 1988 (28)	22	0	2	7
Huang 2020 (31)	158	3	3	32
Jackson 1989 (33)	38	8	21	53
Janssen 1994 (34)	65	1	3	33
Kamal 2009 (35)	33	2	2	3
Masaryk 1987 (36)	8	2	1	9
Modic 1986 (37)	28	7	4	23
Mullin 2000 (38)	20	0	1	10
Mullin 2000 (38)	20	0	1	10
Mullin 2000 (38)	20	1	1	9
Mullin 2000 (38)	20	1	1	9
Mullin 2000 (38)	19	0	2	10
Mullin 2000 (38)	18	0	3	10
Thornbury 1993 (40)	68	10	6	11

### Heterogeneity test

**Figure 4:**
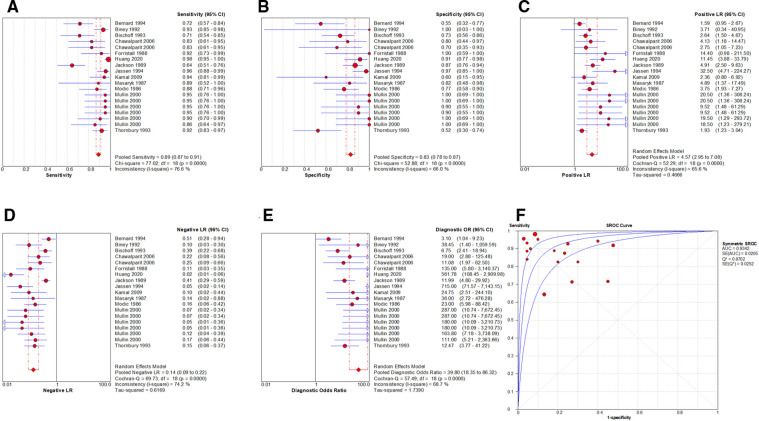
Forest plot of MRI for the diagnosis of LDH. The subgraph of (**A–F**) refers to Sen, Spe, +LR, −LR, DOR, AUC and Q*, respecti**v**ely.

By Spearman's correlation analysis, the correlation coefficient between the logarithm of Sen and the logarithm of (1−Spe) was −0.394, *P* = 0.095, indicating that there was no threshold effect in this meta-analysis. The heterogeneity test results showed that the heterogeneity of Sen (*χ*^2^ = 77.02, *P* = 0.000, *I*^2^ = 76.6%), Spe (*χ*^2^ = 52.88, *P* = 0.000, *I*^2^ = 66.0%), +LR (Cochran-*Q* = 52.29, *P* = 0.000, *I*^2^ = 65.6%), −LR (Cochran-*Q* = 69.73, *P* = 0.000, *I*^2^ = 74.2%), and DOR (Cochran-*Q* = 57.49, *P* = 0.001, *I*^2^ = 68.7%) among the studies were high (Figure 4). The cause of heterogeneity was not found through meta-regression or subgroup analysis. Therefore, the effect sizes were pooled using a random effects model.

### Evaluation index of diagnostic test

The effect sizes of Sen_(pooled)_, Spe_(pooled)_, +LR_(pooled)_, −LR_(pooled)_, DOR_(pooled)_, AUC of SROC, and Q* were 0.89 (95%CI: 0.87–0.91), 0.83 (95%CI: 0.78–0.87), 4.57 (95%CI: 2.95–7.08), 0.14 (95%CI: 0.09–0.22), 39.80 (95%CI: 18.35–86.32), 0.934, and 0.870, respectively (Figure 4).

### Sensitivity analysis and publication bias analysis

**Figure 5:**
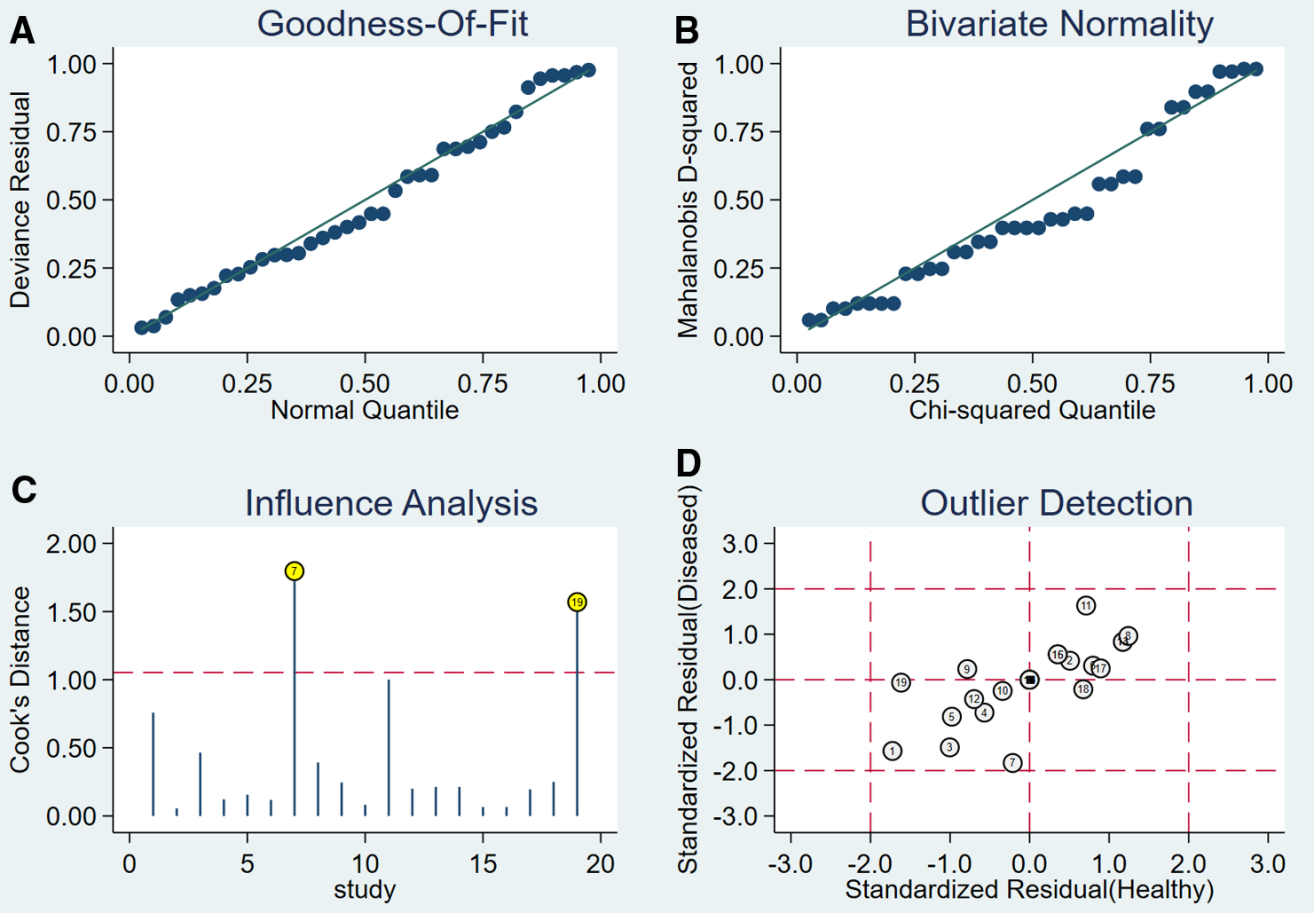
The sensitivity analysis of MRI.

**Figure 6:**
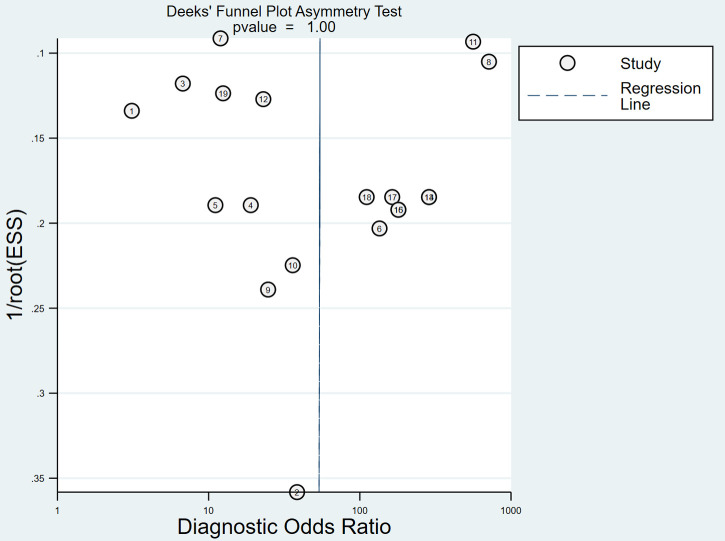
Funnel plot of MRI for the diagnosis of LDH.

After the exclusion of individual studies one by one, the remaining studies were pooled and analyzed again. The results showed that each excluded study had a minor impact on the amount of pooling effect, indicating that the results of this meta-analysis were stable and reliable (Figure 5). Funnel plot was drawn with the inverse of the square root of the effective sample size (ESS) as the ordinate and DOR as the abscissa. The results the slope coefficient was 1.00, suggesting that there was no publication bias (Figure 6).

### Meta-analysis of CT

Ten articles with 10 studies were included (Table 3).

**Table 3:** Characteristics of CT diagnostic tests.

Study name	TP	FP	FN	TN
Firooznia 1984 (27)	97	4	8	7
Forristall 1988 (28)	20	2	4	5
Gillström 1986 (29)	28	1	0	2
Haughton 1982 (30)	29	8	1	17
Huang 2020 (31)	156	5	5	30
Jackson 1989 (32)	89	25	36	81
Jackson 1989 (33)	35	8	24	53
Modic 1986 (37)	25	5	4	19
Schipper 1987 (39)	140	8	57	30
Thornbury 1993 (40)	17	5	1	9

### Heterogeneity test

**Figure 7:**
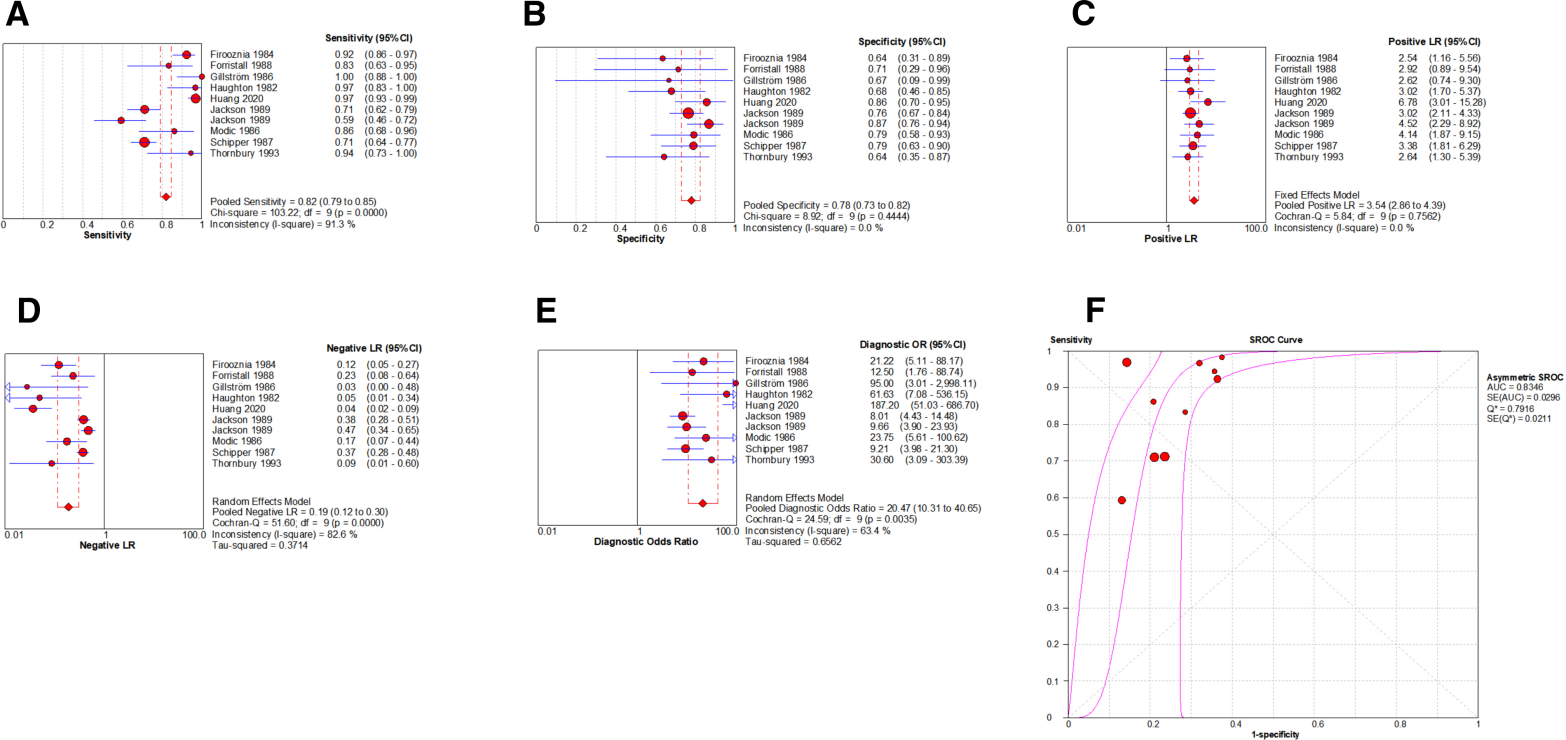
Forest plot of CT for the diagnosis of LDH. The subgraph of (**A–F**) refers to Sen, Spe, +LR, −LR, DOR, AUC and Q*, respecti**v**ely.

By Spearman's correlation analysis, the correlation coefficient between the logarithm of Sen and the logarithm of (1−Spe) was 0.539, *P* = 0.108, indicating that there was no threshold effect in this meta-analysis. The heterogeneity test results showed that the heterogeneity of Sen (*χ*^2^ = 103.22, *P* = 0.000, *I*^2^ = 91.3%), −LR (Cochran-*Q* = 51.60, *P* = 0.000, *I*^2^ = 82.6%), and DOR (Cochran-*Q* = 24.59, *P* = 0.004, *I*^2^ = 63.4%) among the studies were high. The cause of heterogeneity was not found by meta-regression or subgroup analysis, so the random effects model were used for pooling. The heterogeneity of Spe (*χ*^2^ = 8.92, *P* = 0.444, *I*^2^ = 0.0%) and +LR (Cochran-*Q* = 5.84, *P* = 0.756, *I*^2^ = 0.0%) among the studies were low (Figure 7). Therefore, the effect sizes were pooled using a fixed effects model.

### Evaluation index of diagnostic test

The effect sizes of Sen_(pooled)_, Spe_(pooled)_, +LR_(pooled)_, −LR_(pooled)_, DOR_(pooled)_, AUC of SROC, and Q* were 0.82 (95%CI: 0.79–0.85), 0.78 (95%CI: 0.73–0.82), 3.54 (95%CI: 2.86–4.39), 0.19 (95%CI: 0.12–0.30), 20.47 (95%CI: 10.31–40.65), 0.835, and 0.792, respectively (Figure 7).

### Sensitivity analysis and publication bias analysis

**Figure 8:**
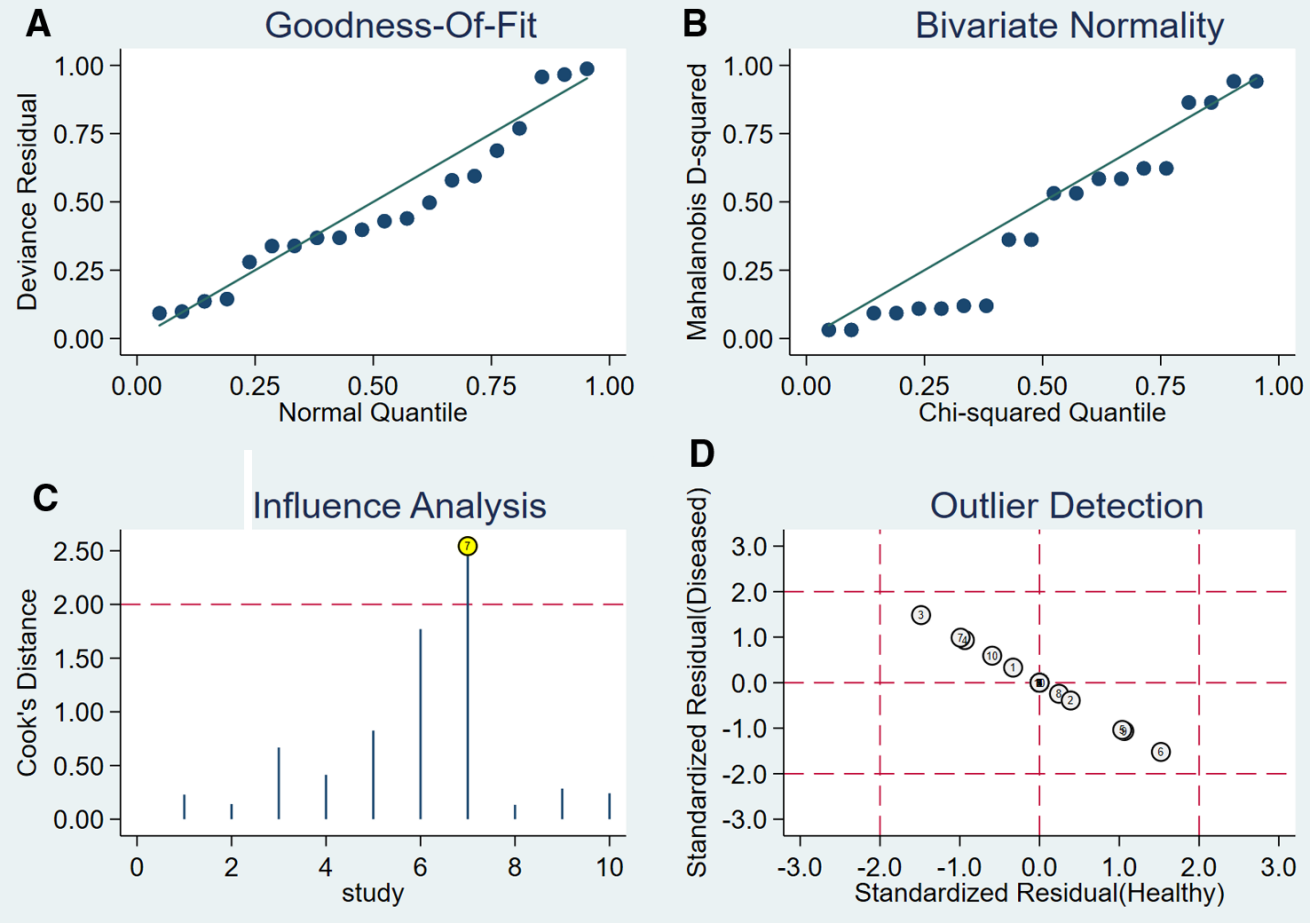
The sensitivity analysis of CT.

**Figure 9:**
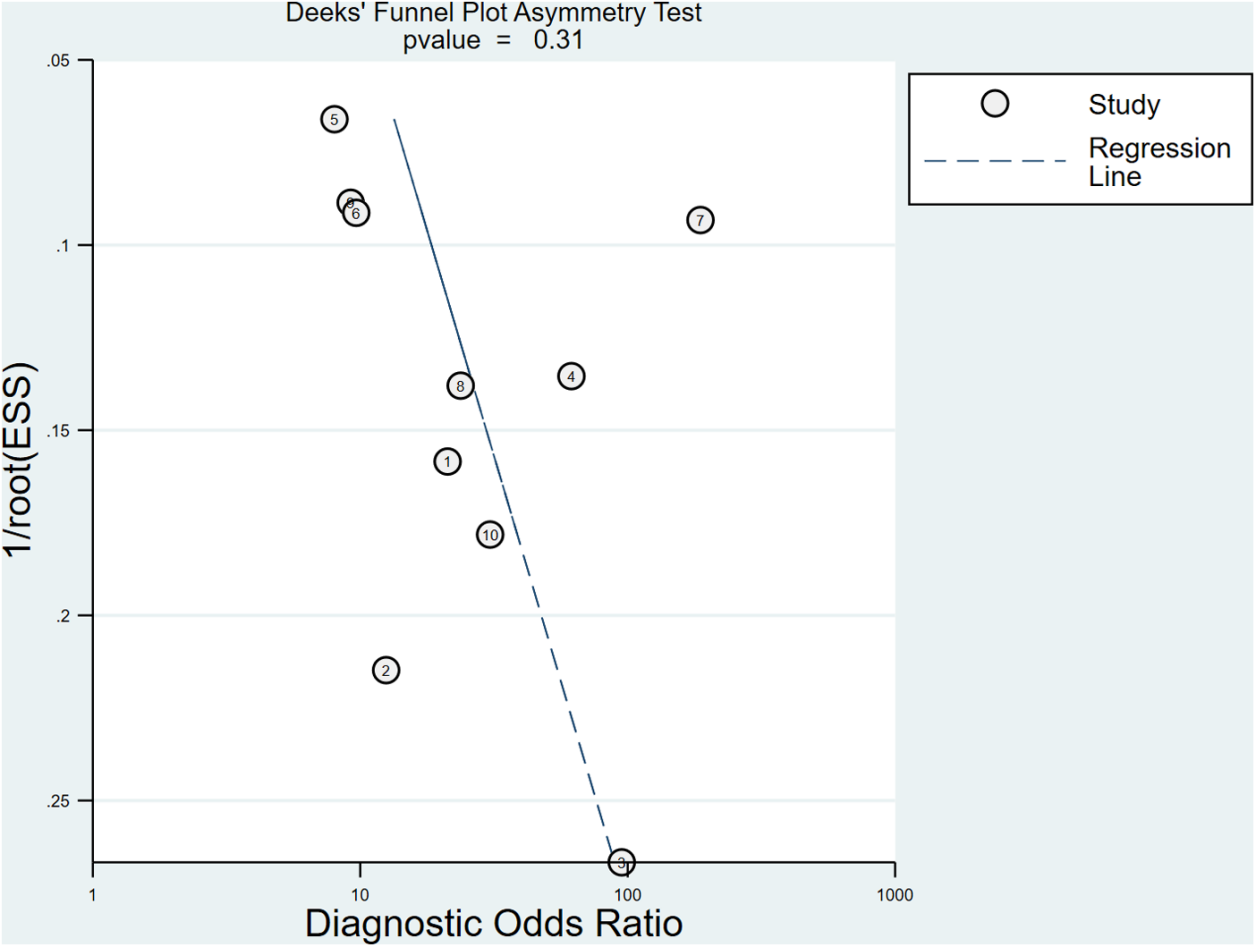
Funnel plot of CT for the diagnosis of LDH.

After the exclusion of individual studies one by one, the remaining studies were pooled and analyzed again. The results showed that each excluded study had a minor impact on the amount of pooling effect, indicating that the results of this meta-analysis were stable and reliable (Figure 8). Funnel plot was drawn with 1/root (ESS) as the ordinate and DOR as the abscissa. The results showed that the *P*-value of the slope coefficient was 0.31, suggesting that there was no publication bias (Figure 9).

### Meta-analysis of myelography

Nine articles with nine studies were included (Table 4).

**Table 4:** Characteristics of myelography diagnostic tests.

Study name	TP	FP	FN	TN
Aejmelaeus 1984 (22)	91	5	8	20
Bischoff 1993 (25)	19	4	16	33
Gillström 1986 (29)	21	1	5	2
Haughton 1982 (30)	28	9	2	16
Jackson 1989 (32)	88	32	37	74
Jackson 1989 (33)	33	8	26	53
Janssen 1994 (34)	55	7	13	27
Modic 1986 (37)	27	11	5	13
Schipper 1987 (39)	191	10	38	24

### Heterogeneity test

**Figure 10:**
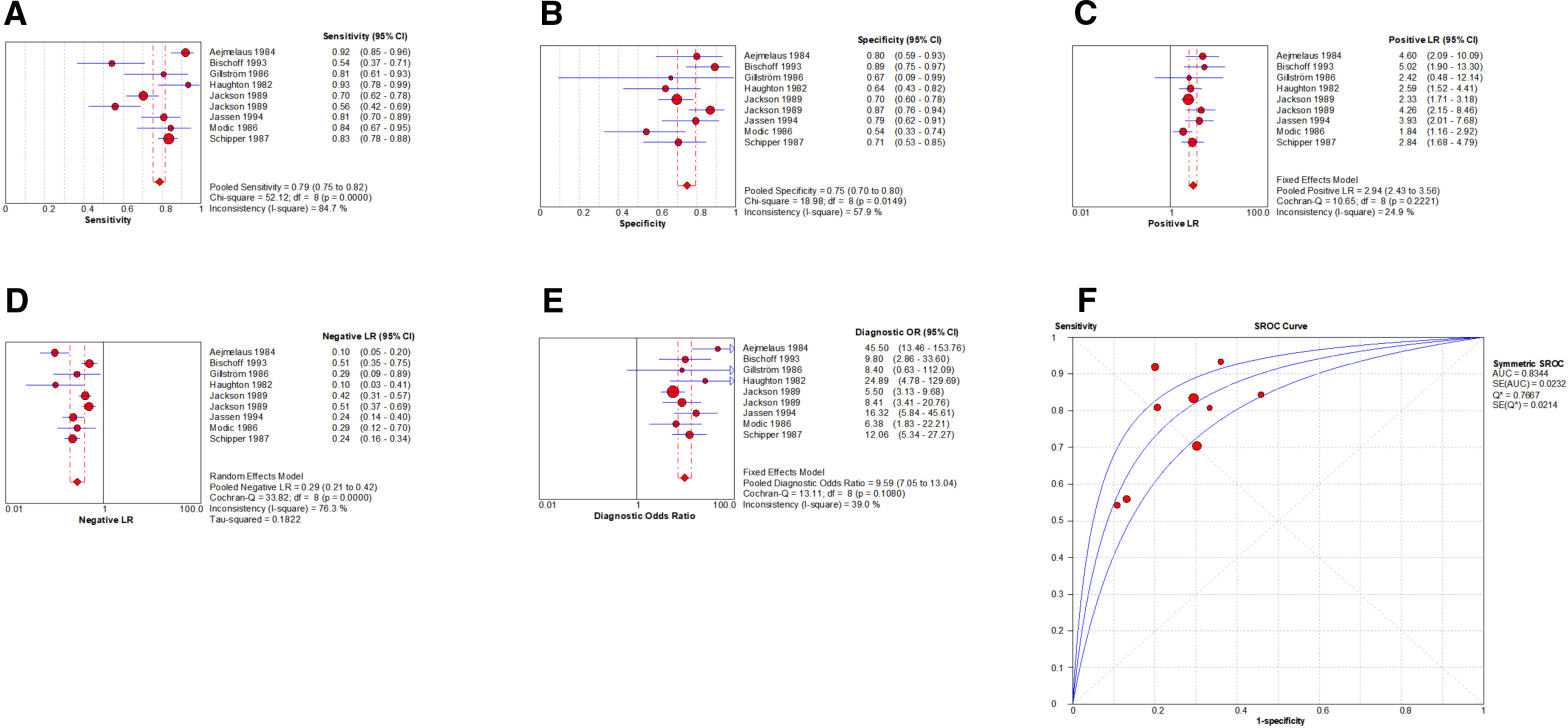
Forest plot of myelography for the diagnosis of LDH. The subgraph of (**A–F**) refers to Sen, Spe, +LR, −LR, DOR, AUC and Q*, respecti**v**ely.

By Spearman's correlation analysis, the correlation coefficient between the logarithm of Sen and the logarithm of (1−Spe) was 0.583, *P* = 0.099, indicating that there was no threshold effect in this meta-analysis. The heterogeneity test results showed that the heterogeneity of Sen (*χ*^2^ = 52.12, *P* = 0.000, *I*^2^ = 84.7%), Spe (*χ*^2^ = 18.98, *P* = 0.015, *I*^2^ = 57.9%), and –LR (Cochran-*Q* = 33.82, *P* = 0.000, *I*^2^ = 76.3%) among the studies were high. The cause of heterogeneity was not found by meta-regression or subgroup analysis, so the effect sizes were pooled using a random effects model. The heterogeneity of +LR (Cochran-*Q* = 10.65, *P* = 0.222, *I*^2^ = 24.9%) and DOR (Cochran-*Q* = 13.11, *P* = 0.108, *I*^2^ = 39.0%) among the studies were low (Figure 10). Therefore, the effect sizes were pooled using a fixed effects model.

### Evaluation index of diagnostic test

The effect sizes of Sen_(pooled)_, Spe_(pooled)_, +LR_(pooled)_, −LR_(pooled)_, DOR_(pooled)_, AUC of SROC, and Q* were 0.79 (95%CI: 0.75–0.82), 0.75 (95%CI: 0.70–0.80), 2.94 (95%CI: 2.43–3.56), 0.29 (95%CI: 0.21–0.42), 9.59 (95%CI: 7.05–13.04), 0.834, and 0.767, respectively (Figure 10).

### Sensitivity analysis and publication bias analysis

**Figure 11:**
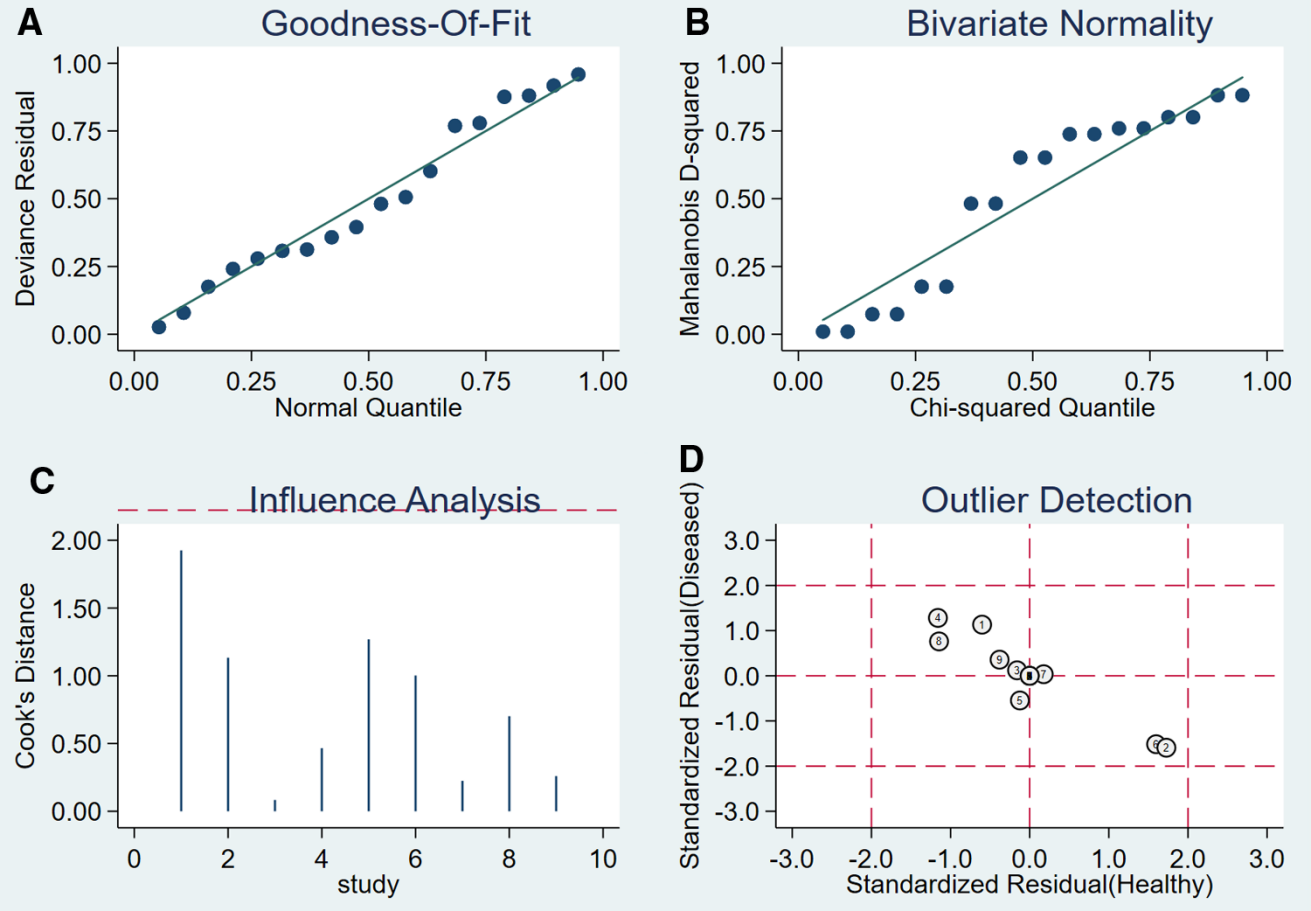
The sensitivity analysis of myelography.

**Figure 12:**
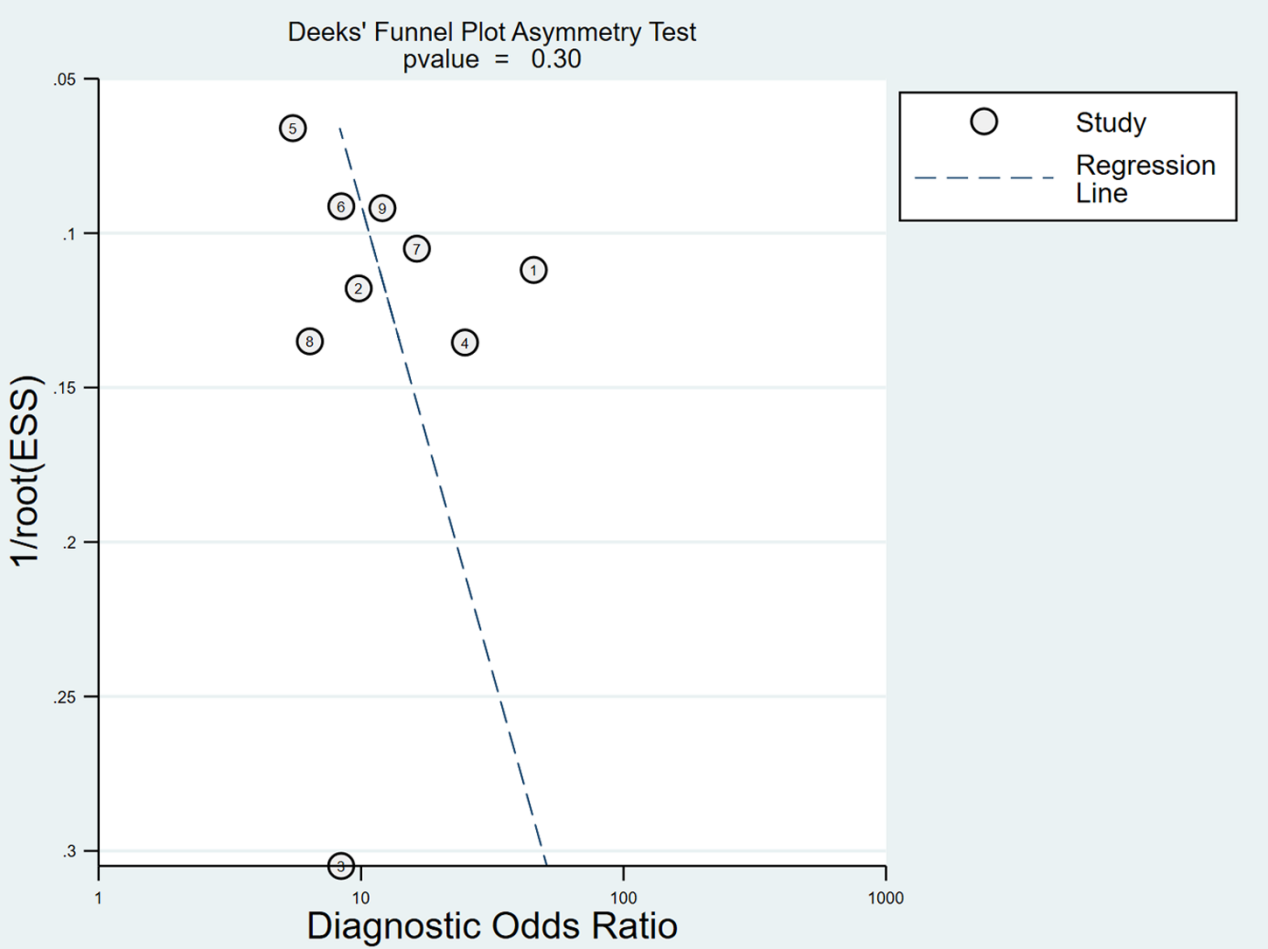
Funnel plot of myelography for the diagnosis of LDH.

After the exclusion of individual studies one by one, the remaining studies were pooled and analyzed again. The results showed that each excluded study had a minor impact on the amount of pooling effect, indicating that the results of this meta-analysis were stable and reliable (Figure 11). Funnel plot was drawn with 1/root (ESS) as the ordinate and DOR as the abscissa. The results showed that the *P*-value of the slope coefficient was 0.30, suggesting that there was no publication bias (Figure 12).

## Discussion

Imaging examinations have important clinical significance for the diagnosis and treatment of LDH. They can provide not only a basis for diagnosis but also a basis for choosing conservative treatment or surgical treatment and surgical methods (42) so as to improve the treatment level. At present, the commonly used imaging examinations include MRI, CT, myelography, and x-ray. MRI is the most established of the imaging examinations, as it has the advantage of not using ionizing radiation and has good visualizing capacities, especially for soft tissue (43). MRI can also comprehensively observe whether each lumbar intervertebral disc has lesions, identify the degree and location of nucleus pulposus herniation on the sagittal plane, and distinguish whether there are other space-occupying lesions in the spinal canal. CT can show the shape of the bony spinal canal and the size and direction of intervertebral disc herniation. It has great diagnostic value for this disease. At present, CT is being commonly used (44). Compared with MRI, CT has the advantages of low cost, shorter total testing time, and larger availability of CT scanners in hospital settings but has the disadvantage of exposure to ionizing radiation. Myelography requires an injection of a contrast medium when testing, under specific circumstances (e.g., metal implant or malalignment of the spine). Myelography can replace MRI as the imaging examination (45). An x-ray cannot directly identify the existence of LDH. Scoliosis, vertebral marginal hyperplasia, and narrowing of intervertebral space on the film all suggest degenerative changes. If the lumbosacral structure is abnormal (e.g., transitional spine, spondylolisthesis, and spondylolysis), it indicates that the adjacent intervertebral discs will accelerate the degeneration and increase the chance of protrusion owing to the increase of stress. With the development of technology in recent times, an x-ray examination is rarely used at present (46).

The comparison of effect sizes showed that the pooled Sen of MRI [0.89 (95%CI: 0.87–0.91)] was higher than that of myelography [0.79 (95%CI: 0.75–0.82)]. The pooled DOR of MRI [39.80 (95%CI: 18.35–86.32)] was also higher than that of myelography [9.59 (95%CI: 7.05–13.04)].

To improve the stability and reliability of the research results, during the implementation of this meta-analysis, two reviewers independently extracted data and evaluated the risk of bias in the included studies. Strict inclusion criteria and exclusion criteria were formulated during literature screening. Considering the differences between studies, the effect sizes with high heterogeneity were analyzed by meta-regression and subgroup analysis. But the source of heterogeneity was not found, the random effects model was used for pooling. Sensitivity and publication bias analyses were performed to make the final results more reliable. However, because of the differences in the condition of patients, medical equipment, and the doctors' proficiency in imaging examination, the heterogeneity of some effect sizes could be high, and it was difficult to find the source of heterogeneity. Finally, some studies regarded patients as research objects, while others regarded lumbar discs as research objects, which also affect the results of this meta-analysis.

## Conclusion

MRI, CT, and myelography have a high value in the diagnosis of LDH; however, the diagnostic value of MRI is higher than that of myelography. Therefore, reasonable selection should be made in combination with the patients' condition.

## Data Availability

The original contributions presented in the study are included in the article/**Supplementary Materials**, further inquiries can be directed to the corresponding author/s.

## References

[1] Fardon DF Milette PC. Nomenclature and classification of lumbar disc pathology. Recommendations of the combined task forces of the North American spine society, American society of spine radiology, and American society of neuroradiology. Spine. (2001) 26(5):E93–113. 10.1097/00007632-200103010-00006 11242399

[2] Vialle LR Vialle EN Suárez Henao JE Giraldo G. Lumbar disc herniation. Rev Bras Ortop. (2015) 45(1):17–22. 10.1016/S2255-4971(15)30211-1 27019834 PMC4799068

[3] Jordan J Konstantinou K O'Dowd J. Herniated lumbar disc. BMJ Clin Evid. (2009) 2009:1118. 19445754 PMC2907819

[4] Lurie J Tomkins-Lane C. Management of lumbar spinal stenosis. Br Med J. (2016) 352:h6234. 10.1136/bmj.h6234 26727925 PMC6887476

[5] Deyo RA Rainville J Kent DL. What can the history and physical examination tell us about low back pain? JAMA. (1992) 268(6):760–5. 10.1001/jama.1992.03490060092030 1386391

[6] Jarvik JG Deyo RA. Diagnostic evaluation of low back pain with emphasis on imaging. Ann Intern Med. (2002) 137(7):586–97. 10.7326/0003-4819-137-7-200210010-00010 12353946

[7] de Schepper EI Koes BW Veldhuizen EF Oei EH Bierma-Zeinstra SM Luijsterburg PA. Prevalence of spinal pathology in patients presenting for lumbar MRI as referred from general practice. Fam Pract. (2016) 33(1):51–6. 10.1093/fampra/cmv097 26659653

[8] Takashima H Takebayashi T Yoshimoto M Terashima Y Ida K Yamashita T. Efficacy of diffusion-weighted magnetic resonance imaging in diagnosing spinal root disorders in lumbar disc herniation. Spine. (2013) 38(16):E998–1002. 10.1097/BRS.0b013e31829862d3 23632334

[9] Bossuyt PM Reitsma JB Bruns DE Gatsonis CA Glasziou PP Irwig L STARD 2015: an updated list of essential items for reporting diagnostic accuracy studies. Br Med J. (2015) 351:h5527. 10.1136/bmj.h5527 26511519 PMC4623764

[10] Page MJ McKenzie JE Bossuyt PM Boutron I Hoffmann TC Mulrow CD The PRISMA 2020 statement: an updated guidelince for reporting systematic reviews. Br Med J. (2021) 372:n71. 10.1136/bmj.n71 33782057 PMC8005924

[11] Whiting PF Rutjes AW Westwood ME Mallett S Deeks JJ Reitsma JB QUADAS-2: a revised tool for the quality assessment of diagnostic accuracy studies. Ann Intern Med. (2011) 155(8):529–36. 10.7326/0003-4819-155-8-201110180-00009 22007046

[12] Sun B Song Q Zhang H Zhang X Luo Y Lu Z Diagnostic performance of magnetic resonance imaging for colorectal liver metastasis:a meta-analysis. J Clin Radiol. (2021) 40(3):516–21. 10.13437/j.cnki.jcr.2021.03.023

[13] Du M Zhang X Zhang Y. Laparoscopic exploration in the diagnosis of tuberculous peritonitis: a meta-analysis. Chin J Evid-Based Med. (2020) 20(1):40–6. 10.7507/1672-2531.201907072

[14] Huedo-Medina TB Sánchez-Meca J Marín-Martínez F Botella J. Assessing heterogeneity in meta-analysis: Q statistic or *I*^2^ index? Psychol Methods. (2006) 11(2):193–206. 10.1037/1082-989X.11.2.193 16784338

[15] De J Yang L Wang Y. Des-γ-carboxy prothrombin in the diagnosis of primary hepatocellular carcinoma: a systematic review. Chin J Evid-Based Med. (2020) 20(7):798–808. 10.7507/1672-2531.201909033

[16] Devillé WL Buntinx F Bouter LM Montori VM de Vet HC van der Windt DA Conducting systematic reviews of diagnostic studies: didactic guidelines. BMC Med Res Methodol. (2002) 2:9. 10.1186/1471-2288-2-9 12097142 PMC117243

[17] Gallagher EJ. Clinical utility of likelihood ratios. Ann Emerg Med. (1998) 31(3):391–7. 10.1016/s0196-0644(98)70352-x 9506499

[18] Glas AS Lijmer JG Prins MH Bonsel GJ Bossuyt PM. The diagnostic odds ratio: a single indicator of test performance. J Clin Epidemiol. (2003) 56(11):1129–35. 10.1016/s0895-4356(03)00177-x 14615004

[19] Mitchell MD. Validation of the summary ROC for diagnostic test meta-analysis: a Monte Carlo simulation. Acad Radiol. (2003) 10(1):25–31. 10.1016/s1076-6332(03)80784-5 12529025

[20] Gao L Xie Y Jia C Wang W. Prevalence of depression among Chinese university students: a systematic review and meta-analysis. Sci Rep. (2020) 10(1):15897. 10.1038/s41598-020-72998-1 32985593 PMC7522998

[21] Khatami F Saatchi M Zadeh SST Aghamir ZS Shabestari AN Reis LO A meta-analysis of accuracy and sensitivity of chest CT and RT-PCR in COVID-19 diagnosis. Sci Rep. (2020) 10(1):22402. 10.1038/s41598-020-80061-2 33372194 PMC7769992

[22] Aejmelaeus R Hiltunen H Härkönen M Silfverhuth M Vähä-Tahlo T Tunturi T. Myelographic versus clinical diagnostics in lumbar disc disease. Arch Orthop Trauma Surg (1978). (1984) 103(1):18–25. 10.1007/BF00451314 6466060

[23] Bernard TN Jr. Using computed tomography/discography and enhanced magnetic resonance imaging to distinguish between scar tissue and recurrent lumbar disc herniation. Spine. (1994) 19(24):2826–32. 10.1097/00007632-199412150-00017 7899986

[24] Birney TJ White JJ Jr Berens D Kuhn G. Comparison of MRI and discography in the diagnosis of lumbar degenerative disc disease. J Spinal Disord. (1992) 5(4):417–23. 10.1097/00002517-199212000-00006 1490039

[25] Bischoff RJ Rodriguez RP Gupta K Righi A Dalton JE Whitecloud TS. A comparison of computed tomography-myelography, magnetic resonance imaging, and myelography in the diagnosis of herniated nucleus pulposus and spinal stenosis. J Spinal Disord. (1993) 6(4):289–95. 10.1097/00002517-199306040-00002 8219542

[26] Chawalparit O Churojana A Chiewvit P Thanapipatsir S Vamvanij V Charnchaowanish P. The limited protocol MRI in diagnosis of lumbar disc herniation. J Med Assoc Thai. (2006) 89(2):182–9. 16579004

[27] Firooznia H Benjamin V Kricheff II Rafii M Golimbu C. CT Of lumbar spine disk herniation: correlation with surgical findings. AJR Am J Roentgenol. (1984) 142(3):587–92. 10.2214/ajr.142.3.587 6607651

[28] Forristall RM Marsh HO Pay NT. Magnetic resonance imaging and contrast CT of the lumbar spine. Comparison of diagnostic methods and correlation with surgical findings. Spine. (1988) 13(9):1049–54. 10.1097/00007632-198809000-00013 3206299

[29] Gillström P Ericsson K Hindmarsh T. A comparison of computed tomography and myelography in the diagnosis of lumbar disc herniation. Arch Orthop Trauma Surg (1978). (1986) 106(1):12–4. 10.1007/BF00435644 3566489

[30] Haughton VM Eldevik OP Magnaes B Amundsen P. A prospective comparison of computed tomography and myelography in the diagnosis of herniated lumbar disks. Radiology. (1982) 142(1):103–10. 10.1148/radiology.142.1.7053518 7053518

[31] Huang M Wu L Kuang X Peng W. Applied research of auxiliary diagnostic system of CT image enhancement in the diagnostic of LDH. China Med Eq. (2020) 17(12):44–8. 10.3969/J.ISSN.1672-8270.2020.12.011

[32] Jackson RP Becker GJ Jacobs RR Montesano PX Cooper BR McManus GE. The neuroradiographic diagnosis of lumbar herniated nucleus pulposus: I. A comparison of computed tomography (CT), myelography, CT-myelography, discography, and CT-discography. Spine. (1989) 14(12):1356–61. 10.1097/00007632-198912000-00012 2694388

[33] Jackson RP Cain JE Jr Jacobs RR Cooper BR McManus GE. The neuroradiographic diagnosis of lumbar herniated nucleus pulposus: II. A comparison of computed tomography (CT), myelography, CT-myelography, and magnetic resonance imaging. Spine. (1989) 14(12):1362–7. 10.1097/00007632-198912000-00013 2694389

[34] Janssen ME Bertrand SL Joe C Levine MI. Lumbar herniated disk disease: comparison of MRI, myelography, and post-myelographic CT scan with surgical findings. Orthopedics. (1994) 17(2):121–7. 10.3928/0147-7447-19940201-07 8190676

[35] Kamal F Quddus M Hossain A Rahman M Sarkar R Nabi S Role of magnatic resonance imaging (MRI) in the pre-operative diagnosis of lumbar disc herniation. J Dhaka Med Coll. (2009) 18(1):8–14. 10.3329/jdmc.v18i1.6298

[36] Masaryk TJ Ross JS Modic MT Boumphrey F Bohlman H Wilber G. High-resolution MR imaging of sequestered lumbar intervertebral disks. AJR Am J Roentgenol. (1988) 150(5):1155–62. 10.2214/ajr.150.5.1155 3258720

[37] Modic MT Masaryk T Boumphrey F Goormastic M Bell G. Lumbar herniated disk disease and canal stenosis: prospective evaluation by surface coil MR, CT, and myelography. AJR Am J Roentgenol. (1986) 147(4):757–65. 10.2214/ajr.147.4.757 3489378

[38] Mullin WJ Heithoff KB Gilbert TJ Jr Renfrew DL. Magnetic resonance evaluation of recurrent disc herniation: is gadolinium necessary? Spine. (2000) 25(12):1493–9. 10.1097/00007632-200006150-00007 10851097

[39] Schipper J Kardaun JW Braakman R van Dongen KJ Blaauw G. Lumbar disk herniation: diagnosis with CT or myelography. Radiology. (1987) 165(1):227–31. 10.1148/radiology.165.1.3628775 3628775

[40] Thornbury JR Fryback DG Turski PA Javid MJ McDonald JV Beinlich BR Disk-caused nerve compression in patients with acute low-back pain: diagnosis with MR, CT myelography, and plain CT. Radiology. (1993) 186(3):731–8. 10.1148/radiology.186.3.8267688 8267688

[41] Tian J Chen Y Yang K Song F. Progresses and challenges for meta analysis or systematic review. J Lanzhou Univ (Med Sci). (2016) 42(1):42–7. 10.13885/j.issn.1000-2812.2016.01.008

[42] Tsai MD Jou SB Hsieh MS. A new method for lumbar herniated inter-vertebral disc diagnosis based on image analysis of transverse sections. Comput Med Imaging Graph. (2002) 26(6):369–80. 10.1016/s0895-6111(02)00033-2 12453503

[43] Wassenaar M van Rijn RM van Tulder MW Verhagen AP van der Windt DA Koes BW Magnetic resonance imaging for diagnosing lumbar spinal pathology in adult patients with low back pain or sciatica: a diagnostic systematic review. Eur Spine J. (2012) 21(2):220–7. 10.1007/s00586-011-2019-8 21922287 PMC3265584

[44] Lurie JD. What diagnostic tests are useful for low back pain? Best Pract Res Clin Rheumatol. (2005) 19(4):557–75. 10.1016/j.berh.2005.03.004 15949776

[45] Pomerantz SR. Myelography: modern technique and indications. Handb Clin Neurol. (2016) 135:193–208. 10.1016/B978-0-444-53485-9.00010-6 27432666

[46] Yu X Niu G Yang J Ni L Zhang W Guo Y. Quantitative evaluation for diagnostic efficacy of computed tomography and magnetic resonance imaging in patients with lumbar disc herniation. Natl Med J China. (2011) 91(1):23–7. 10.3760/cma.j.issn.0376-2491.2011.01.007 21418957

