# Supplementary material for: Value of imaging examinations in diagnosing lumbar disc herniation: A systematic review and meta-analysis
Source: Front Surg. 2023 Jan 6;9:1020766. doi: 10.3389/fsurg.2022.1020766 (PMC9872518; doi:10.3389/fsurg.2022.1020766)
Supplement: Supplementary file 1 [file Table1.doc]

**SUPPLEMENTARY METHOD S1** Electronic search strategies

**PubMed**

#1 ((((((((((lumbar disc herniation[Title/Abstract]) OR (lumbar herniated disc[Title/Abstract])) OR (lumber disc herniation[Title/Abstract])) OR (protrusion of intervertebral disc[Title/Abstract])) OR (PID[Title/Abstract])) OR (prolapsed lumbar intervertebral disc[Title/Abstract])) OR (lumbar intervertebral disc herniation[Title/Abstract])) OR (lumbar intervertebral disc protrusion[Title/Abstract])) OR (prolapse of lumbar intervertebral disc[Title/Abstract])) OR (lumbar disc protrusion[Title/Abstract])) OR (LHD[Title/Abstract])

#2 (((((Computed Tomography[Title/Abstract]) OR (CT[Title/Abstract])) OR (Magnetic Resonance[Title/Abstract])) OR (Magnetic Resonance Imaging[Title/Abstract])) OR (MRI[Title/Abstract]) OR (Myelography[Title/Abstract])

#3 sensitiv*[Title/Abstract] OR sensitivity and specificity[MeSH Terms] OR (predictive[Title/Abstract] AND value*[Title/Abstract]) OR predictive value of tests[Title/Abstract] OR accuracy*[Title/Abstract]

#4 #1 AND #2 AND #3

Note: We searched 148 articles while using PubMed as the filter.

**Embase**

#1 'lumbar disc herniation':ab,ti OR 'lumbar herniated disc':ab,ti OR 'lumber disc herniation':ab,ti OR 'protrusion of intervertebral disc':ab,ti OR 'PID':ab,ti OR 'prolapsed lumbar intervertebral disc':ab,ti OR 'lumbar intervertebral disc herniation':ab,ti OR 'lumbar intervertebral disc protrusion':ab,ti OR 'prolapse of lumbar intervertebral disc':ab,ti OR 'lumbar disc protrusion':ab,ti OR 'LDH':ab,ti

#2 'Computed Tomography':ab,ti OR 'CT':ab,ti OR 'Magnetic Resonance':ab,ti 0R 'magnetic resonance imaging':ab,ti OR 'MRI':ab,ti OR 'Myelography':ab,ti

#3 'sensitiv':ab,ti OR 'sensitivity and specificity':ab,ti OR 'predictive':ab,ti OR 'predictive value of tests':ab,ti OR 'accuracy':ab,ti

#4 #1 AND #2 AND #3

Note: We searched 129 articles while using RefMan-(RIS) as the filter.

**Cochrane Library**

#1 (lumbar disc herniation):ab,ti,kw OR (lumbar herniated disc):ab,ti,kw OR (lumber disc herniation):ab,ti,kw OR (protrusion of intervertebral disc):ab,ti,kw OR (PID):ab,ti,kw OR (prolapsed lumbar intervertebral disc):ab,ti,kw OR (lumbar intervertebral disc herniation):ab,ti,kw OR (lumbar intervertebral disc protrusion):ab,ti,kw OR (prolapse of lumbar intervertebral disc):ab,ti,kw OR (lumbar disc protrusion):ab,ti,kw OR (LHD):ab,ti,kw

#2 (Computed Tomography):ab,ti,kw OR (CT):ab,ti,kw OR (Magnetic Resonance):ab,ti,kw OR (magnetic resonance imaging):ab,ti,kw OR (MRI):ab,ti,kw OR (Myelography):ab,ti,kw

#3 (sensitiv):ab,ti,kw OR (sensitivity and specificity):ab,ti,kw OR (predictive):ab,ti,kw OR (predictive value of tests):ab,ti,kw OR (accuracy):ab,ti,kw

#4 #1 and #2 and #3

Note: We searched 28 articles while using RefMan-(RIS) as the filter.

**Web of Science**

#1 TS=(lumbar disc herniation or lumbar herniated disc or lumber disc herniation or protrusion of intervertebral disc or PID or prolapsed lumbar intervertebral disc or lumbar intervertebral disc herniation or lumbar intervertebral disc protrusion or prolapse of lumbar intervertebral disc or lumbar disc protrusion or LHD)

#2 TS=(Computed Tomography or CT or Magnetic Resonance or magnetic resonance imaging or MRI or Myelography)

#3 TS=(sensitiv or sensitivity and specificity or predictive or predictive value of tests or accuracy)

#4 #1 AND #2 AND #3

Note: We searched 241 articles while using Web of science as the filter.

**CBM**

1 "腰椎间盘"[常用字段:智能] OR "腰椎间盘突出症"[常用字段:智能]

2 "CT"[常用字段:智能] OR "核磁共振"[常用字段:智能] OR "MRI"[常用字段:智能] OR "脊髓造影术"[常用字段:智能] OR "椎管造影术"[常用字段:智能]

3 "诊断"[常用字段:智能]

4 1 AND 2 AND 3

Note: We searched 3743 articles while using SinoMed as the filter.

**CNIK**

1 腰椎间盘突出 OR 腰椎间盘突出

2 CT OR 核磁共振 OR MRI OR 脊髓造影术 OR 椎管造影术

3 诊断

4 1 AND 2 AND 3

Note: We searched 1595 articles while using NoteExpress as the filter.

**WanFang Data**

主题:(腰椎间盘突出 or 腰椎间盘突出症) and 主题:(CT or 核磁共振 or MRI or脊髓造影术 or 椎管造影术) and (诊断)

Note: We searched 1067 articles while using NoteExpress as the filter.

**VIP**

#1 腰椎间盘突出+腰椎间盘突出症

#2 CT+核磁共振+MRI+脊髓造影术+椎管造影术

#3 诊断

#4 #1与#2与#3

Note: We searched 1007 articles while using NoteExpress as the filter.
