# Supplementary material for: Value of imaging examinations in diagnosing lumbar disc herniation: A systematic review and meta-analysis
Source: Front Surg. 2023 Jan 6;9:1020766. doi: 10.3389/fsurg.2022.1020766 (PMC9872518; doi:10.3389/fsurg.2022.1020766)
Supplement: Supplementary file 3 [file Table3.docx]

**TABLE 3 |** Characteristics of CT diagnostic tests.

| **Study name** | **TP** | **FP** | **FN** | **TN** |
| --- | --- | --- | --- | --- |
| Firooznia 1984 (27) | 97 | 4 | 8 | 7 |
| Forristall 1988 (28) | 20 | 2 | 4 | 5 |
| Gillström 1986 (29) | 28 | 1 | 0 | 2 |
| Haughton 1982 (30) | 29 | 8 | 1 | 17 |
| Huang 2020 (31) | 156 | 5 | 5 | 30 |
| Jackson 1989 (32) | 89 | 25 | 36 | 81 |
| Jackson 1989 (33) | 35 | 8 | 24 | 53 |
| Modic 1986 (37) | 25 | 5 | 4 | 19 |
| Schipper 1987 (39) | 140 | 8 | 57 | 30 |
| Thornbury 1993 (40) | 17 | 5 | 1 | 9 |
