# Supplementary material for: Value of imaging examinations in diagnosing lumbar disc herniation: A systematic review and meta-analysis
Source: Front Surg. 2023 Jan 6;9:1020766. doi: 10.3389/fsurg.2022.1020766 (PMC9872518; doi:10.3389/fsurg.2022.1020766)
Supplement: Supplementary file 4 [file Table4.docx]

**TABLE 4 |** Characteristics of Myelography diagnostic tests.

| **Study name** | **TP** | **FP** | **FN** | **TN** |
| --- | --- | --- | --- | --- |
| Aejmelaus 1984 (22) | 91 | 5 | 8 | 20 |
| Bischoff 1993 (25) | 19 | 4 | 16 | 33 |
| Gillström 1986 (29) | 21 | 1 | 5 | 2 |
| Haughton 1982 (30) | 28 | 9 | 2 | 16 |
| Jackson 1989 (32) | 88 | 32 | 37 | 74 |
| Jackson 1989 (33) | 33 | 8 | 26 | 53 |
| Jassen 1994 (34) | 55 | 7 | 13 | 27 |
| Modic 1986 (37) | 27 | 11 | 5 | 13 |
| Schipper 1987 (39) | 191 | 10 | 38 | 24 |
