# Supplementary material for: Value of imaging examinations in diagnosing lumbar disc herniation: A systematic review and meta-analysis
Source: Front Surg. 2023 Jan 6;9:1020766. doi: 10.3389/fsurg.2022.1020766 (PMC9872518; doi:10.3389/fsurg.2022.1020766)
Supplement: Supplementary file 6 [file Table6.doc]

SUPPLEMENTAL TABLE 1 | Details of quality assessment by the QUADAS-2 tool.

| **references** | **22** | **23** | **24** | **25** | **26** | **27** | **28** | **29** | **30** | **31** | **32** | **33** | **34** | **35** | **36** | **37** | **38** | **39** | **40** |
| --- | --- | --- | --- | --- | --- | --- | --- | --- | --- | --- | --- | --- | --- | --- | --- | --- | --- | --- | --- |
| Was a consecutive or random sample of patients enrolled? | √ | √ | √ | √ | √ | √ | √ | √ | √ | √ | √ | √ | √ | √ | √ | √ | √ | √ | √ |
| Was a case-control design avoided? | √ | √ | √ | √ | √ | √ | √ | √ | √ | √ | √ | √ | √ | √ | √ | √ | √ | √ | √ |
| Did the study avoid inappropriate exclusions? | √ | √ | √ | √ | √ | √ | √ | √ | √ | √ | √ | √ | √ | √ | √ | √ | √ | √ | √ |
| Are there concerns that the included patients and setting do not match the review question? | L | L | L | L | L | L | L | L | L | L | L | L | L | L | L | L | L | L | L |
| Were the index test results interpreted without knowledge of the results of the reference standard? | √ | √ | √ | √ | √ | √ | √ | √ | √ | ? | √ | √ | √ | √ | √ | √ | √ | √ | √ |
| If a threshold was used, was it pre-specified? | √ | √ | √ | √ | √ | √ | √ | √ | √ | √ | √ | √ | √ | √ | √ | √ | √ | √ | √ |
| Are there concerns that the index test, its conduct, or interpretation differ from the review question? | L | L | L | L | L | L | L | L | L | L | L | L | L | L | L | L | L | L | L |
| Is the reference standards likely to correctly classify the target condition? | √ | √ | √ | √ | √ | √ | √ | √ | √ | √ | √ | √ | √ | √ | √ | √ | √ | √ | √ |
| Were the reference standard results interpreted without knowledge of the results of the index tests? | × | × | × | × | × | × | × | × | × | ? | × | × | × | × | × | × | × | × | × |
| Are there concerns that the target condition as defined by the reference standard does not match the question? | L | L | L | L | L | L | L | L | L | L | L | L | L | L | L | L | L | L | L |
| Was there an appropriate interval between index test and reference standard? | ? | ? | √ | ? | ? | ? | ? | ? | ? | ? | √ | ? | ? | √ | ? | ? | ? | ? | ? |
| Did all patients receive the same reference standard? | √ | √ | √ | √ | √ | √ | √ | √ | √ | √ | √ | √ | √ | √ | √ | √ | √ | √ | √ |
| Were all patients included in the analysis? | × | √ | × | × | × | √ | × | √ | × | √ | √ | √ | √ | √ | √ | √ | √ | × | × |

Abbreviations**:** √=yes; ×=no; ?=unclear;L=low risk;U=unclear risk;H=high risk
